# Combination of immunosuppressive therapy and nintedanib improves capillaroscopic changes in systemic sclerosis-interstitial lung disease: a case report

**DOI:** 10.1093/rap/rkac003

**Published:** 2022-02-10

**Authors:** Shogo Matsuda, Mahiro Yamamoto, Takuya Kotani, Tohru Takeuchi

**Affiliations:** Department of Internal Medicine (IV), Osaka Medical and Pharmaceutical University, Takatsuki, Osaka, Japan

Key message

- Combination of immunosuppressive therapy and nintedanib may modulate microvascular changes in SSc-interstitial lung disease.

Dear Editor, SSc is a complex autoimmune disease that involves systemic organs, such as skin, heart, intestine and lung. Interstitial lung disease (ILD) is a major pulmonary complication and is related to poor prognosis [1]. Nailfold videocapillaroscopy (NVC) is a useful tool for evaluating microvascular abnormalities in SSc [2]. These microvascular abnormalities are defined as scleroderma pattern, which is included in the 2013 ACR/EULAR classification criteria for SSc [3]. This scleroderma pattern can be divided into early, active and late patterns. These patterns are correlated with the disease duration of SSc, and a natural progression of microvascular damage is observed from the early pattern to the late pattern. NVC is also a candidate tool for detecting SSc-ILD, and microscopic changes, such as a severe pattern and progression of capillary loss, are useful indicators that have helped clinicians to develop a better screening algorithm for SSc [2]. However, there were few reports investigating the association between NVC findings and therapeutic response in SSc-ILD. In this study, we provide the first case report of a patient with SSc-ILD who showed a reversible change in NVC findings after a combination of immunosuppressive therapy with nintedanib.

A 71-year-old woman was admitted to our hospital with a history of worsening dyspnoea. (New York Heart Association class II). On examination, she had RP and telangiectasia in both hands. She did not have sclerodactyly. Serological tests for ANA and anti-ribonucleoprotein antibody were positive. ILD was diagnosed on chest high-resolution CT (HRCT; Fig. 1A). Her percentage forced vital capacity was within normal limits. Echocardiogram was within normal limits, indicating no sign of pulmonary hypertension. NVC was then performed to evaluate microvascular damage. We examined two adjacent fields of 1 mm in the middle of the nailfold of all fingers of both hands except the thumbs, as referenced in a previous study [4]. NVC revealed giant capillaries, frequent haemorrhages and reduced capillary density, which are seen in the active pattern of SSc (Fig. 1B).

**Fig. 1:**
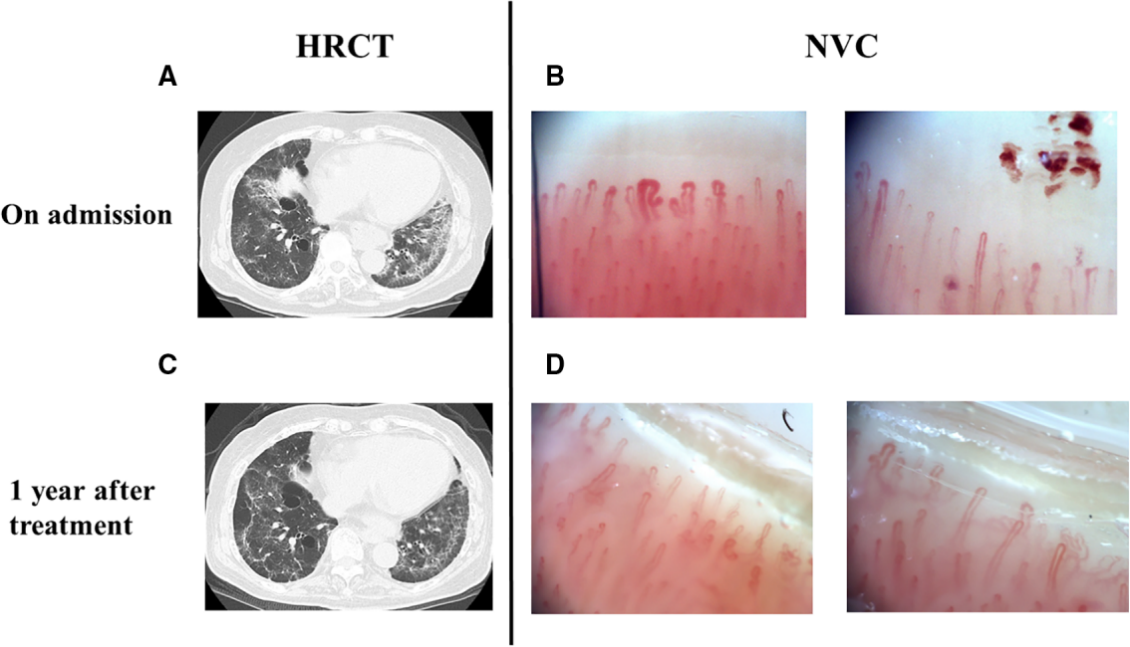
Nailfold videocapillaroscopy findings and chest high-resolution CT images in SSc-interstitial lung disease (**A**) Chest high-resolution CT (HRCT) image on admission. Ground-glass opacities are apparent bilaterally in the lower lung lobes. (**B**) Nailfold videocapillaroscopy (NVC) findings on admission. Giant capillaries and haemorrhage can be seen. Magnification ×200. (**C**) Chest HRCT images at 12 months after the intervention. There is a reduction in ground-glass opacities in the left lower lung lobe. (**D**) NVC findings at 12 months after the intervention. The number of microhaemorrhages has decreased markedly, and giant capillaries have disappeared. Magnification ×200.

She was diagnosed with SSc, based on the presence of RP, ILD, abnormal nailfold capillary and telangiectasia in both hands, according to the 2013 ACR criteria [3]. In HRCT findings, her ILD affected >10% of the lung volume, and the ground-glass opacities on chest HRCT showed a gradual increase in comparison to 6 months previously. The serum KL-6 level was 2813 U/ml (range 105–401 U/ml) on admission. Given that ILD had progressed sub-acutely, immunosuppressive therapy with prednisolone (25 mg daily) and tacrolimus (1 mg daily), and nintedanib (200 mg daily) were initiated on day 14.

After initiation of the combination therapy, the patient’s ILD improved without any additional exacerbations during this course (Fig. 1C). At 12 months after the start of induction therapy, the patient’s serum KL-6 levels had decreased to 394 U/l, the number of microhaemorrhages had markedly decreased, and giant capillaries had disappeared on NVC. Also, the number of capillaries was ≥7 capillaries/mm. Furthermore, the NVC pattern of SSc changed from an active pattern to normal [5] (Fig. 1D).

Previous reports showed that immunosuppressive treatments, such as haematopoietic stem-cell transplantation, CYC and iloprost, improved NVC findings in SSc patients [6]. However, few reports have shown modification of the microvascular architecture after treatment in SSc patients. In our patient, we found that the combination of immunosuppressive therapy and nintedanib modulated the microvascular damage in SSc-ILD.

In SSc, several growth factors, including VEGF and PDGF, are related to microvascular damage [7]. Nintedanib, a tyrosine kinase inhibitor, specifically inhibits PDGF receptors, fibroblast growth factor receptors and VEGF receptors. Nintedanib also inhibits the proliferation of several vascular cell types in the lung, such as endothelial cells and smooth muscle cells [8]. In idiopathic pulmonary fibrosis model mice, nintedanib improves the distorted microvascular architecture of the lung [8]. Based on these findings, nintedanib might inhibit vasculogenesis of nailfold capillaries by suppressing growth factors, leading to an improvement in the microvascular architecture. In addition to nintedanib, immunosuppressive therapy might improve microvascular damage by inhibiting microvascular inflammation in SSc-ILD [1]. Whether nintedanib or immunosuppressive therapy contributed to the improvement of microvasculopathy should be examined by accumulating future cases.

A previous study showed that rapid progression to the late pattern in NVC was associated with further clinical manifestations of SSc [2]. Also, it was reported that presence of ILD was higher in SSc patients with the late pattern than in those with the early and active pattern [9]. In our patient, her NVC finding was the active pattern and percentage forced vital capacity was within the normal range on admission; therefore, her ILD was thought to be detected in the early phase [10]. Our report suggests that the combination of immunosuppressive therapy and nintedanib is more likely to suppress vascular damage in the early phase of SSc-ILD than in the late phase.

In summary, we report a case in which a combination of immunosuppressive therapy and nintedanib was effective for nailfold microvascular damage in SSc-ILD. NVC findings were improved in parallel with the improvement of SSc-ILD; therefore, longitudinal assessment of NVC findings might serve as a useful tool to evaluate response to treatment of SSc-ILD. Further studies are needed to clarify the pathomechanism of immunosuppressive therapy and nintedanib therapy in treating microvascular damage in SSc-ILD.

*Funding:* No specific funding was received from any bodies in the public, commercial or not-for-profit sectors to carry out the work described in this article.

*Disclosure statement:* The authors have declared no conflicts of interest.

*Patient consent:* Written informed consent was obtained from the patient for publication of this case report.

## Data availability statement

The data underlying this article will be shared on reasonable request to the corresponding author.
